# Introduction of a team-based care model in a general medical unit

**DOI:** 10.1186/s12913-016-1507-2

**Published:** 2016-07-11

**Authors:** Stephanie E. Hastings, Esther Suter, Judy Bloom, Krishna Sharma

**Affiliations:** Alberta Health Services, 10301 Southport Lane SW, Calgary, AB T2W 1S7 Canada

**Keywords:** Collaborative care, Collaborative practice, Model of care, Patient outcomes, Provider outcomes, Evaluation, Evidence-based practice, Patient-centred care, Family-centred care

## Abstract

**Background:**

Alberta Health Services is a provincial health authority responsible for healthcare for more than four million people. The organization recognized a need to change its care delivery model to make care more patient- and family-centred and use its health human resources more effectively by enhancing collaborative practice. A new care model including changes to how providers deliver care and skill mix changes to support the new processes was piloted on a medical unit in a large urban acute care hospital Evidence-based care processes were introduced, including an initial patient assessment and orientation, comfort rounds, bedside shift reports, patient whiteboards, Name Occupation Duty, rapid rounds, and team huddles. Small teams of nurses cared for a portion of patients on the unit. The model was intended to enhance safety and quality of care by allowing providers to work to full scope in a collaborative practice environment.

**Methods:**

We evaluated the new model approximately one year after implementation using interviews with staff (*n* = 15), surveys of staff (*n* = 25 at baseline and at the final evaluation) and patients (*n* = 26 at baseline and 37 at the final evaluation), and administrative data pulled from organizational databases.

**Results:**

Staff interviews revealed that overall, the new care processes and care teams worked quite well. Unit culture and collaboration were improved, as were role clarity, scope of practice, and patient care. Responses from staff surveys were also very positive, showing significant positive changes in most areas. Patient satisfaction surveys showed a few positive changes; scores overall were very high. Administrative data showed slight decreases in overall length of stay, 30-day readmissions, staff absenteeism, staff vacancies, and the overtime rate. We found no changes in unit length of stay, 30-day returns to emergency department, or nursing sensitive adverse events.

**Conclusions:**

Conclusions from the evaluation were positive, providing initial support for the idea of the collaborative practice model vision for adult medical units across Alberta. There were also a few positive effects on patient care suggesting that models such as this one could improve the organization’s ability to deliver sustainable, high-quality, patient- and family-centred care without compromising quality.

## Background

Emerging evidence that lack of communication and collaboration between healthcare providers can seriously harm patients, reduce patient satisfaction, and lead to duplication and inefficiencies has driven healthcare organizations to focus on finding solutions [1]. One way in which organizations have tried to meet these demands is through interprofessional collaboration, where different professional groups work together to positively impact healthcare [2]. The introduction of collaborative practice models was prompted by research showing that interprofessional collaboration improves quality of care and patient outcomes [2, 3].

At the same time, rising costs and overall demand to improve quality and safety have placed increasing pressure on these organizations to use their human resources more efficiently [4, 5]. Healthcare organizations also need to properly organize and deploy health care providers to improve their collective ability to work to full scope of practice and achieve high quality patient care. Alberta Health Services (AHS) is particularly conscious of the need to have the right provider, in the right place, for the right patient due to the size and mandate of the organization.

AHS is a provincial health authority responsible for providing care to more than four million people. It is Canada’s first province-wide health system and employs more than 100 000 staff, making it one of the largest employers in the country [6]. AHS has 105 acute care hospitals comprising over 8000 acute or sub-acute care beds, and a budget of nearly 14 billion dollars annually. The provincial population is growing rapidly and patients’ needs are becoming more complex; AHS recognized a need to change its care delivery model and its vision of professional practice to ensure the health system is sustainable and capable of meeting the increasing needs of the province.

A new initiative was developed to enhance the care experience and health outcomes of Albertans by introducing collaborative practice and optimizing the skill mix in adult medical and surgical inpatient units to foster transformational clinical change. The goal of the model was not to simply change the provider mix but to introduce “a new way of operating and relating to all health providers and most importantly patients and their families” ([7], p.3). Patient- and family-centred care was at the core of the model, necessitating an emphasis on information sharing, participation, and collaboration with patients and families [8].

The model involved changes to how providers deliver care and staffing changes to support the new care processes. The new care processes were based on leading practices and were identified through literature searches, environmental scans, simulations, and interviews with patients, staff, and physicians. All were aimed at improving the care experience and increasing patients’ and families’ engagement by making the care more patient- and family-centred. The new care processes introduced were:

- Name Occupation Duty (NOD): Every provider in contact with a patient introduces him- or herself, states their role, and states the duty they are to perform for the patient;
- Initial patient assessment and orientation: A Registered Nurse (RN) or Licensed Practical Nurse (LPN) conducts a patient assessment within 60 min of arrival to the unit and provides verbal information about various aspects of unit functioning;
- Comfort rounding: Health Care Aides (HCA) visit patients’ rooms at least once every two hours to check pain, positioning, toileting, and that patients have everything they need within reach;
- Bedside shift report: RNs report to each other at each patient’s bedside at every shift change and conduct a number of safety checks. This time is also used to allow patients and families to ask questions or raise concerns;
- Patient whiteboards: Each patient has a whiteboard near his or her bed with providers’ names, a list of the day’s appointments, care goals, anticipated date of discharge, and space for patients and families to leave messages for staff;
- Rapid rounds: Physicians, nurses, and allied health staff hold daily interprofessional rounds to discuss patients’ care plan, anticipated date of discharge, and barriers to discharge;
- Assignment of care and care hub huddles: RNs assign duties to members of the care team and teams meet (“huddle”) regularly throughout the shift to reassess the plan for the day.

To better support the new care processes, the basis of nursing care at the unit level was reorganized into small teams (“care hubs”) of providers (RNs, LPNs, and HCAs) who care for a portion of the patients on the unit. One RN served as the care hub lead on each shift, overseeing (on day shift) one to two LPNs and HCAs. Staffing varied by shift, with fewer staff on evening and night shifts. Care hub leads were responsible for coordinating the work of the rest of the team, communicating with the team about patient status, and caring for more acute patients. During the implementation phase of the project, an additional resource - the Collaborative Practice Lead - was available around the clock to help guide the staff to practice within the new model and offer additional support to the team when necessary. Collaborative Practice Leads observed staff performing the new care processes using a detailed checklist and provided feedback on fidelity. The unit manager and nurse educator also supported the new model by observing processes and providing feedback.

The new model was implemented in September 2013 on one general medical unit (Unit A) of a large hospital in one of Alberta’s two large urban centres following detailed education and training on the new care processes and care hubs. The goal of the current study was to evaluate outcomes of the model for patients, providers and the healthcare system approximately one year after implementation. We used patient and provider surveys, provider interviews, and patient and human resources outcomes data pulled from AHS administrative databases.

## Methods

This was an evaluation of an internal initiative and therefore did not require ethics approval. However, we sought a privacy impact assessment and complied with all relevant health information regulations and ethics guidelines throughout the course of the project.

### Staff interviews

We conducted semi-structured interviews with nursing and allied health staff on Unit A, as well as a manager and a physician (*n* = 15; nine nursing providers, four allied health providers, one manager, and one physician). Interviews began with an affirmation of consent to be interviewed and for the interview to be recorded. We recorded the interviews and took detailed notes. Interview guides focused on teamwork and collaboration, the new care practices, role clarity, quality of care, and thoughts about the model in general. We used realist thematic analysis [9] to analyze the interviews based on the notes, using the recordings to check accuracy where necessary. All results were validated by a second analyst.

### Staff surveys

Staff surveys measured staff perceptions of quality of patient care, manager support of staff and change, role clarity, time and autonomy, scope, support, engagement, and collaboration and communication with colleagues, as well as intention to turnover. We developed the staff survey for this project based on a literature review and the Canadian Interprofessional Health Collaborative [10] national interprofessional competency framework. We selected a total of 32 items for administration. Perceptions were measured with likert scale (1 = strongly disagree to 5 = strongly agree) items. The exception was intention to leave position in the next 12 months, which was measured with Yes or No responses. Staff completed surveys at baseline and the final evaluation. Cronbach’s alpha for the full survey was 0.94; Cronbach’s alphas for each subscale are shown in Table 1.

**Table 1:** Staff survey response frequencies

	Strongly disagree (1)	Disagree (2)	Neither agree nor disagree (3)	Agree (4)	Strongly agree (5)	N/A
Quality of Patient Care (α = .83)						
My co-workers are committed to doing quality work	0	1 (4.0)	3 (12.0)	14 (56.0)	7 (28.0)	0
My colleagues and I work effectively together to help patients	0	0	2 (8.0)	13 (52.0)	10 (40.0)	0
The quality of patient care on my last shift was excellent	0	0	5 (20.0)	14 (56.0)	6 (24.0)	0
I am confident my colleagues provide good patient care	0	0	3 (12.0)	13 (52.0)	9 (36.0)	0
I receive the appropriate patient information from my colleagues at the right time	0	2 (8.0)	2 (8.0)	13 (52.0)	8 (32.0)	0
Manager Support of Staff & Change (α = .93)						
The unit manager supports active quality assurance programs	0	0	1 (4.0)	9 (36.0)	15 (60.0)	0
The unit manager is supportive of staff	0	0	1 (4.0)	9 (36.0)	15 (60.0)	0
The unit manager expects high standards of patient care	0	0	2 (8.0)	7 (28.0)	16 (64.0)	0
The manager/supervisor communicates well with the rest of the unit	0	1 (4.0)	0	9 (36.0)	15 (60.0)	0
I feel valued and affirmed by the unit manager	0	0	2 (8.0)	8 (32.0)	15 (60.0)	0
The unit manager is committed to supporting change	0	0	0	8 (32.0)	17 (68.0)	0
The unit manager supports innovative ideas about delivery of patient care	0	0	0	9 (36.0)	8 (32.0)	0
Role Clarity (α = .83)						
My colleagues from other disciplines have a good understanding of the distinction between my role and their roles	0	0	5 (20.0)	14 (56.0)	6 (24.0)	0
My colleagues from other disciplines have a good understanding of my role	0	0	5 (20.0)	14 (56.0)	6 (24.0)	0
I have a good understanding of the roles of my colleagues from other disciplines	0	0	4 (16.0)	12 (48.0)	9 (36.0)	0
Time & Autonomy (α = .84)						
I have sufficient time to spend in “value-added” patient care activities including comprehensive assessment (that is, biomedical-psycho-social-cultural-spiritual), health teaching, discharge planning, and patient/family support	1 (4.2)	3 (12.5)	4 (16.7)	12 (50.0)	2 (8.3)	2
There is enough time and opportunity to discuss patient care with other providers	0	1 (4.0)	3 (12.0)	13 (52.0)	8 (32.0)	0
I am satisfied with my involvement in decision making on the unit	0	2 (8.0)	2 (8.0)	12 (48.0)	9 (36.0)	0
I am satisfied with the amount of autonomy I have in my job	0	0	3 (12.0)	11 (44.0)	11 (44.0)	0
Scope (α = .67)						
In my practice setting, I am able to use the full range of knowledge and skills that are associated with my profession	0	1 (4.0)	0	9 (36.0)	15 (60.0)	0
I have the necessary knowledge and skills to do the job	0	0	1 (4.0)	8 (32.0)	16 (64.0)	0
I am clear about what I am expected to accomplish	0	0	1 (4.0)	8 (32.0)	16 (64.0)	0
Support (α = .73)						
I feel valued and affirmed by my colleagues	0	0	1 (4.0)	13 (52.0)	11 (44.0)	0
My team inspires me to do my best work	0	0	1 (4.2)	13 (54.2)	10 (41.7)	0
I feel recognized and appreciated on the unit	0	0	1 (4.0)	13 (52.0)	11 (44.0)	0
The unit colleagues support innovative ideas about delivery of patient care	0	0	4 (16.0)	13 (52.0)	8 (32.0)	0
Engagement (α = .77)						
I am proud to tell others I work for, or with, AHS	0	0	1 (4.0)	5 (20.0)	19 (76.0)	0
My job provides me with a sense of personal accomplishment	0	0	0	9 (37.5)	15 (62.5)	0
I am satisfied with my job	0	0	1 (4.0)	11 (44.0)	13 (52.0)	0
Collaboration/Communication (α = .67)						
Information and knowledge are shared openly within the unit	0	0	2 (8.0)	9 (36.0)	14 (56.0)	0
My colleagues from other disciplines work through conflicts with me in efforts to resolve them	0	0	2 (8.0)	14 (56.0)	9 (36.0)	0
Colleagues from all professional disciplines contribute to developing a shared treatment plan	0	0	1 (4.0)	15 (60.0)	9 (36.0)	0

Note: valid percentages are in parentheses

### Patient surveys

AHS administers the Canadian Patient Experiences Survey – Inpatient Care [11] for all adult inpatient units in the province to measure patient satisfaction. It consists of 55 questions administered by phone to adult patients within six weeks of discharge from an inpatient unit. The survey is administered to approximately 10 % of adult discharges at the hospital level and probes patient experience with various aspects of care using Never, Sometimes, Usually, or Always response options. We selected as indicators for this evaluation 22 questions that focused on patient and family involvement in care decisions, patients’ perceptions of the care experience, and patients’ satisfaction with healthcare services. Patient surveys were also administered at baseline using a previous version of the survey. Where possible, item-level comparisons (based on chi-square analyses combining Never and Sometimes responses versus Usually and Always responses) are provided in the results section. The Canadian Institute for Health Information has extensively tested the full survey for reliability and validity in adult populations in multiple Canadian provinces [12].

### Administrative data

We evaluated eight indicators drawn from a number of administrative databases. The Discharge Abstract Database, Admission Discharge and Transfer, and National Ambulatory Care Reporting Systems, all AHS databases, were the source for all clinical data including patient information, service utilization, and outcomes. Patient data from different databases were linked using hospital, service unit, admission date, discharge date, and unique lifetime identifier (ULI). The ULI was unique to each patient in Alberta and all databases used in this study had ULI as patient identifier. For workforce related indicators, we used the Management Reporting - e-People System database which provided information related to staffing and finance. Patient data were obtained for a 40 month period from September 2011 through to December 2014. Workforce and payroll data were not available for the entire 40 month period due to recent transition of the data from legacy organization systems to a new integrated system. For the most part, workforce data were available for the calendar year of 2012 and onwards. Administrative data indicators were used to assess whether there was a significant change in key measures after the introduction of the new model and whether anything was moving in the wrong direction. We used visual inspection of the trend lines to identify significant changes in trends or spikes. Lowess smoothing of the trend lines was used where applicable to aid the visual analysis. It is important to note that these trend lines alone may not prove the impact of the new model as there could be other factors including seasonal variations which are unaccounted for.

## Results

### Interviews

#### New care processes

For the most part, interviewees thought NOD, whiteboards, comfort rounds, and rapid rounds were going well. Staff felt NOD was helpful for patients with delirium or dementia; these patients were more receptive to their healthcare provider when NOD was used. Interviewees felt the comfort rounds had had a substantial impact on the unit by improving patient care. Patients were happy about knowing when a provider would be returning to the room and rang the call bell less often as a result. Interviewees said rapid rounds worked best when physicians attended, and some noted that because LPNs did not attend rounds, they were not always entirely up to date on patient status. However, rapid rounds were credited with improving communication among providers about plans for patient care.

Bedside shift reports were a challenge at times; staff preferred not to wake patients for report, reports could be lengthy because of the number of patient needs to address during the visit, and privacy in shared rooms was a concern. That said, patients’ families liked the bedside shift report as it gave them a chance to see the healthcare team and ask questions or raise concerns.

Team huddles were helpful for keeping the hubs working as a team rather than slipping back to primary nursing, but were not held consistently by all RNs. Interviewees thought that working as a team to do head-to-toe assessments was helpful, as care needs were easier to attend to, all hub members were informed about the patient, and charting could be completed at the bedside.

#### Care hubs

Unit A initially introduced a two-hub model of care, with a small team of nursing staff (1 RN, 2 LPNs, 1 HCA) caring for half of the patients on the unit (i.e., approximately 16 patients). Staff found this model difficult to follow on day shifts and adjusted the approach to a three-hub model several months after implementation. Each hub consisted of one RN, one LPN, and one HCA, all working together to care for about 10 patients. Night shifts were also technically run as three hubs, but typically worked as a two-hub system with crossover and collaboration between the teams. One hub on the night shift was led by an LPN, and some interviewees thought it would be more effective to have an RN in that role. The move to a three-hub model was because of workload, assignment of care, and concern that patient safety issues could arise in the two-hub model. Opinions varied about which application of the care hub concept was most effective.

An early issue on Unit A was the number of float or relief staff on the unit. Float staff were not accustomed to working in the new model, making it difficult for everyone to work as a team particularly when they were present in proportionally large numbers on the unit for a given shift. As the transformation became more embedded in the unit and float staff were able to be oriented to the new model, the unit was seen to be a preferred place for these staff to work.

#### Unit culture and collaboration

Interviewees thought staff had generally accepted the model, although not all had fully bought into it. There was some agreement that the unit was more collaborative post-implementation and that nursing and allied health staff knew each other better after the changes were introduced. Most interviewees thought communication and overall unit mood was improved in the new model, and it was thought that physicians were able to get information from the RNs as needed. However, interviewees thought hub members only knew about patients they were directly responsible for and not patients in the other hubs, and some noted that care hub members were reluctant to offer help to other hubs.

Work was said to be more organized and efficient in the new model, since providers took on more ownership of their assigned care responsibilities. Within care hubs, team members helped each other out with care whereas in the earlier model, providers spent considerable time trying to find someone who could assist them.

#### Role clarity and scope of practice

Interviewees felt they had a better understanding of each other’s roles since the new model was introduced. LPNs were reported to have more responsibilities and a larger scope in the new model. One interviewee thought nurses were “shining” because they had more accountability for patients. Some said the role of the RN was “heavier” in the new model because they provide leadership and mentorship to the rest of the care hub team and are also responsible for caring for the most acute patients. HCAs were also working to fuller scope, taking on tasks such as taking vital signs and doing basic charting.

#### Patient care

Interviewees agreed that the new model was more patient- and family-centered than the old way of working. RNs were thought to know their patients better, and patients were thought to be happier because they saw care provided in a more predictable way. The comfort rounds, in particular, were thought to improve patient care and satisfaction because they were held regularly and patients knew when the providers would return. Patients were said to be mobilized more often because providers had more time to help them up. Safer discharges and up-to-date communication were also said to have resulted from the new model.

### Staff surveys

The results of the staff survey are shown in Table 1. Twenty five staff members from Unit A completed the survey at each time point. The majority (72 %) were from the nursing group. Most (68 %) worked full time and had been employed by AHS for two to five years (44 %). Only 20 % of participants indicated they were likely to accept another position within the next year, compared with 48 % at baseline.

Responses were very positive for the staff survey. At least 80 % of respondents selected “agree” or “strongly agree” for all but one item. Almost 60 % of respondents felt they had enough time to spend in value-added care such as patient/family support and teaching. Overall, results indicated that respondents were satisfied with quality of patient care, manager support, role clarity, time and autonomy, scope, support, engagement, and collaboration and communication.

**Fig. 1:**
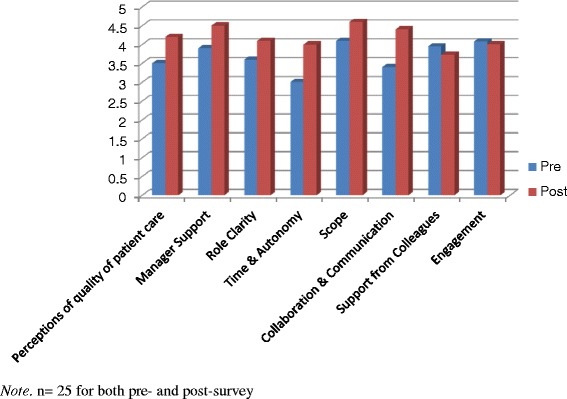
Unit A staff survey pre-post comparisons

We compared the scale means on the final evaluation survey to the baseline survey using independent two-tailed t-tests because we had no way to match baseline and final evaluation surveys and there was a strong likelihood that the surveys were not completed by the same staff members at each time point. We found several significant improvements since the new model was introduced (Fig. 1). Responses increased significantly for perceptions of quality of patient care (4.2 at post-test vs. 3.5 at pre-test; *p* < .001), manager support (4.5 vs. 3.9; *p* = .05), role clarity (4.1 vs. 3.6; *p* < .05), time and autonomy (4.0 vs. 3.0; *p* < .001), scope (4.6 vs. 4.1; *p* < .05), and collaboration and communication (4.4 vs. 3.4; *p* < .001).

A Chi-square test of intent to leave within the next 12 months revealed that Unit A staff were significantly less likely to plan to leave after implementation than they were before the new model was introduced (20 % at final evaluation vs. 48 % at baseline).

### Patient satisfaction surveys

Patient satisfaction survey results were very positive for Unit A (see Table 2 for detailed results; *n* = 37). At least 85 % of patients chose “Usually” or “Always” for most of the items probing patient care experiences. However, just over 20 % of patients said doctors “Never” or “Sometimes” explained things in a way they could understand, and 30 % said staff “Never” or “Sometimes” explained possible side effects of new medications. Almost 50 % of patients said they had not received written information about what side effects to watch for after discharge, and just over 25 % said they “Never” or “Sometimes” received help as soon as they wanted it after pushing the call button.

**Table 2:** Patient satisfaction survey response frequencies

Were you involved as much as you wanted to be in decisions about your care and treatment during this hospital stay?											
Never	3 (8.1)	Sometimes	5 (13.5)	Usually	10 (27.0)	Always	19 (51.4)				
Were your family or friends involved as much as you wanted in decisions about your care and treatment?											
Never	3 (8.3)	Sometimes	2 (5.6)	Usually	5 (13.9)	Always	16 (44.4)	N/A	10 (27.8)		
								Never	Sometimes	Usually	Always
During this hospital stay, how often did nurses treat you with courtesy and respect?								0	4 (10.8)	3 (8.1)	30 (81.1)
During this hospital stay, how often did nurses listen carefully to you?								2 (5.4)	2 (5.4)	6 (16.2)	27 (73.0)
During this hospital stay, how often did nurses explain things in a way you could understand?								2 (5.6)	3 (8.3)	9 (25.0)	22 (61.1)
During this hospital stay, how often did doctors treat you with courtesy and respect?								1 (2.8)	5 (13.9)	7 (19.4)	23 (63.9)
During this hospital stay, how often did doctors listen carefully to you?								1 (2.7)	1 (2.7)	12 (32.4)	23 (62.2)
During this hospital stay, how often did doctors explain things in a way you could understand?								1 (2.8)	7 (19.4)	8 (22.2)	20 (55.6)
During this hospital stay, how often was your pain well controlled?								1 (4.3)	2 (8.7)	9 (39.1)	11 (47.8)
During this hospital stay, how often did the hospital staff do everything they could to help you with your pain?								1 (4.2)	2 (8.3)	5 (20.8)	16 (66.7)
Before giving you any new medicine, how often did hospital staff tell you what the medicine was for?								0	1 (4.5)	1 (4.5)	20 (90.9)
Before giving you any new medicine, how often did hospital staff describe possible side effects in a way you could understand?								3 (15.0)	3 (15.0)	6 (30.0)	8 (40.0)
Do you feel that there was good communication about your care between doctors, nurses and other hospital staff?								2 (5.4)	5 (13.5)	12 (32.4)	18 (48.6)
										No	Yes
During your hospital stay, did doctors, nurses or other hospital staff talk with you about whether you would have the help you needed when you left the hospital?										5 (15.6)	27 (84.4)
During this hospital stay, did you get information, in writing, about what symptoms or health problems to look out for, after you left the hospital?										15 (48.4)	16 (51.6)
							Not at all	Partly	Quite a bit	Completely	N/A
Before you left the hospital, did you have a clear understanding about all your prescribed medication, including those you were taking before your hospital stay?							1 (3.0)	5 (15.2)	3 (9.1)	20 (60.6)	4 (12.1)
After you left the hospital, did you go directly to your own home, to someone else’s home, or to another health facility?											
Own home	32 (91.4)										
Someone else’s home	2 (5.7)										
Another health facility	1 (2.9)										
We want to know your rating of the care you received during this hospital stay. Using any number from 0 to 10 where 0 is the worst possible care and 10 is the best possible care. What number would you give the care you got from all the healthcare providers named below who treated you?											
	Worst possible care	1	2	3	4	5	6	7	8	9	Best possible care
Nurses	1 (2.7)	1 (2.7)	1 (2.7)	0	0	0	5 (13.5)	7 (18.9)	8 (21.6)	4 (10.8)	10 (27.0)
Doctors	1 (2.7)	0	3 (8.1)	1 (2.7)	0	1 (2.7)	2 (5.4)	5 (13.5)	9 (24.3)	4 (10.8)	11 (29.7)
Pharmacists	0	0	0	1 (5.9)	1 (5.9)	0	1 (5.9)	1 (5.9)	2 (11.8)	7 (41.2)	4 (23.5)
							Never	Sometimes	Usually	Always	N/A
During this hospital stay, after you pressed the call button, how often did you get help as soon as you wanted it?							3 (8.6)	6 (17.1)	9 (25.7)	13 (37.1)	4 (11.4)
							Never	Sometimes	Usually	Always	
How often did you get help in getting to the bathroom or in using a bedpan as soon as you wanted?							0	4 (19.0)	6 (28.6)	11 (52.4)	

Note: Valid percentages are in parentheses

Comparisons of baseline (*n* = 26) and final evaluation patient satisfaction scores revealed only a few significant changes. More patients in the post-test felt their family and friends were involved in care (58 % vs. 50 % at baseline), and more patients in the post-test felt providers had told them what any new medications were for (95 % vs. 56 % at baseline).

### Administrative data indicators

**Fig. 2:**
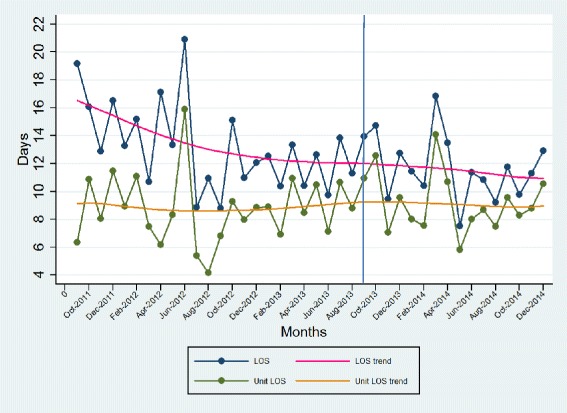
Total and unit length of stay (days)

**Fig. 3:**
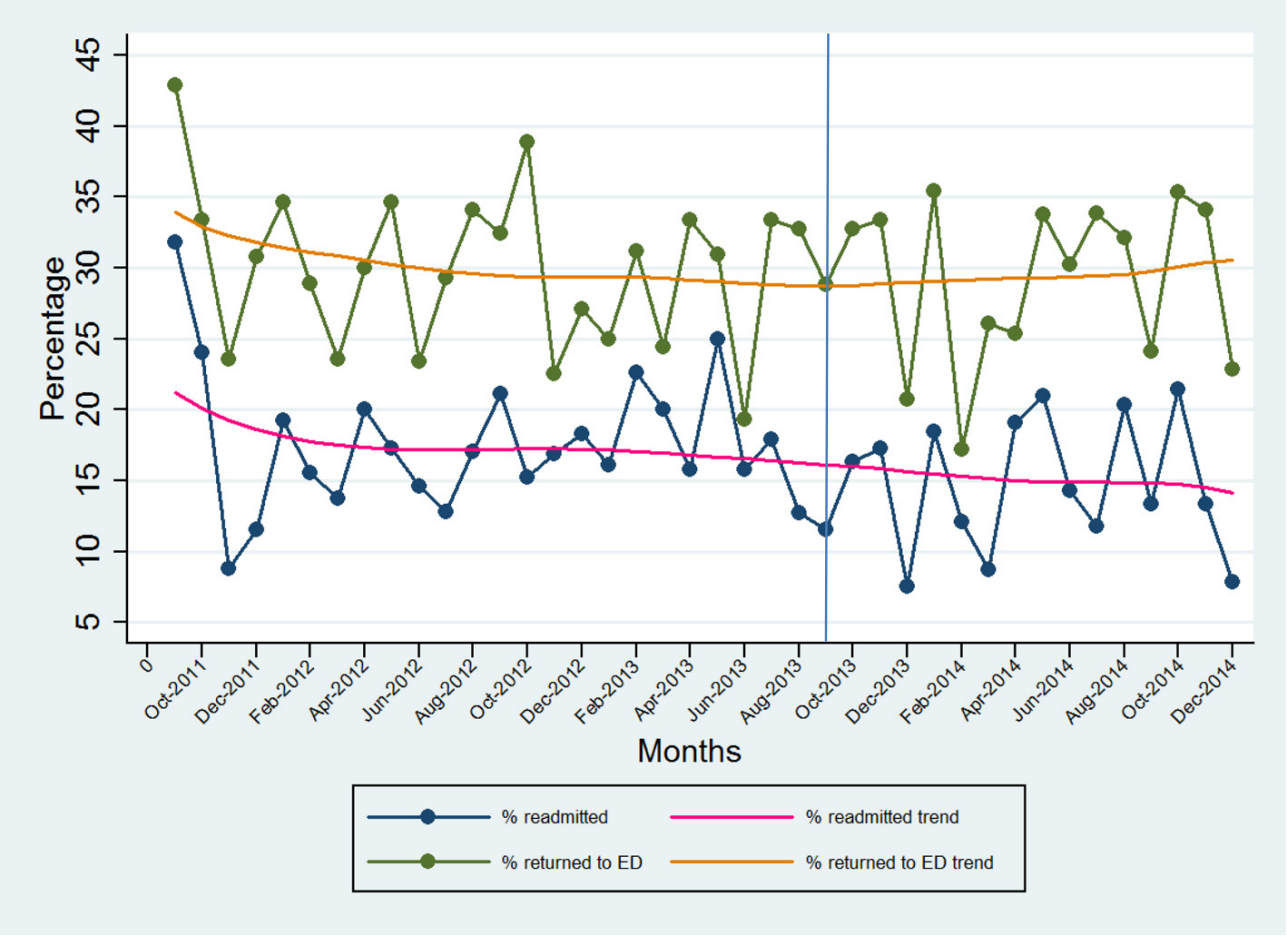
30-day readmission and return to emergency department rates (%)

**Fig. 4:**
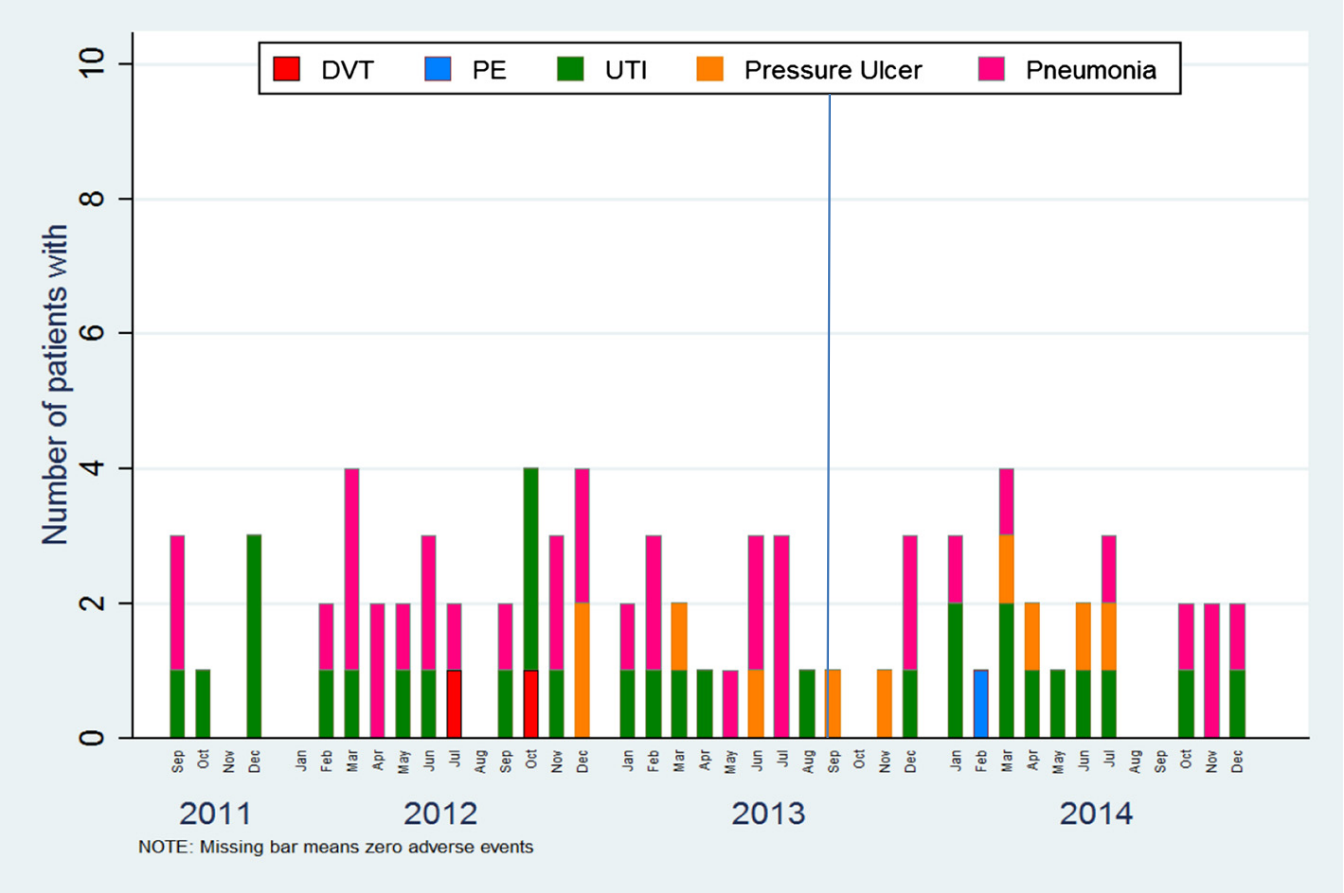
Nursing sensitive adverse events – count of number of patients with any event

Unit A’s detailed results for each of the patient indicators are shown in Figs. 2, 3 and 4. The total length of stay (LOS) showed a decreasing trend over time, whereas the unit LOS trend did not change direction after implementation (Fig. 2). The trend for the 30-day readmission rate showed a slight decline, but the 30 day return to emergency department trend remained stable (Fig. 3). Nursing sensitive adverse event indicators did not show any visible changes in either direction (Fig. 4).

**Fig. 5:**
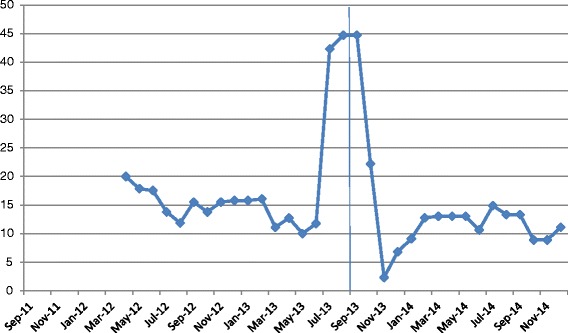
Staff vacancy (%)

**Fig. 6:**
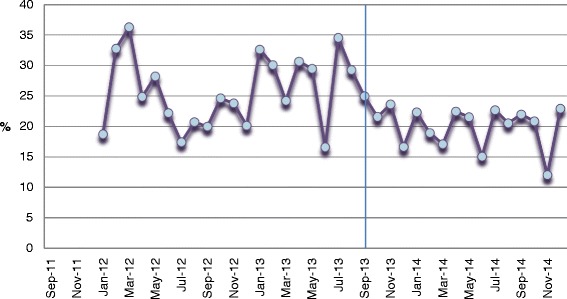
Absenteeism hours rate (%)

**Fig. 7:**
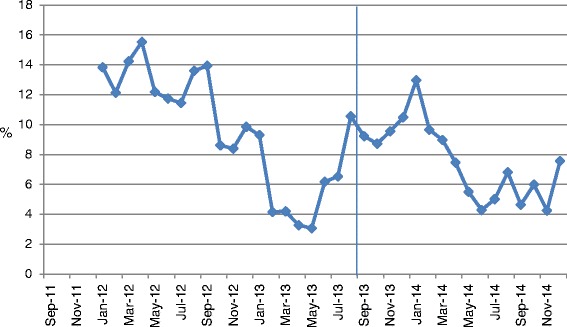
Overtime rate (overtime hours as % of paid hours)

We also evaluated system-level indicators for Unit A, shown in Figs. 5, 6 and 7. The staff vacancy rate saw a declining trend during the post-implementation period although there was a sharp increase in the vacancy rate at the beginning of the implementation (Fig. 5). We observed decreasing trends in the absenteeism rate (Fig. 6) and the overtime rate (Fig. 7).

## Discussion

Conclusions from the evaluation were positive, providing initial support for the idea of the collaborative practice model vision for adult medical units across Alberta. The new collaborative care processes were well-received by staff for the most part and were credited with having improved patient-centred care and patient satisfaction. Providers had more time to spend with patients in the new model and were more able to use their full scope of practice to provide the most appropriate care.

We used multiple data sources to capture outcomes at the patient, provider, and system levels. At the patient level, although there were no significant clinical changes for patients, there were also no detrimental effects. This was important to monitor as the new model was introduced to ensure safety was not compromised as staff learned the new processes. There were only a few significant changes in patient feedback, but staff reported anecdotally that patients seemed more satisfied and needed to use call bells less frequently under the new model.

At the provider level, we found a significant improvement in nearly all components of the staff survey and interviewees reported being relatively pleased with the new model. It was important to include provider reactions in the evaluation in order to give us a sense of the overall mood and culture on the unit. These provider-level findings are consistent with other literature [1] showing that collaborative practice improves workplace quality and staff satisfaction. Engagement of staff is important for retention [13, 14]; our results showed substantially lower turnover intentions among staff on Unit A after implementation. Furthermore, one study [14] found engaged healthcare employees are more likely to report that their work unit provides high quality patient care, has a patient-centred environment, and has a strong culture of patient safety.

System-level indicators did not show significant changes in lengths of stay or admissions, but there were positive changes in the human resources indicators (i.e., vacancy rate, absenteeism, and overtime hours). This suggests that the long-term cost implications for this model are promising.

The evaluation results guide managers to identify specific, intentional efforts that support care providers to work in collaborative care models in optimized roles within a quality professional practice environment. There is good evidence from this evaluation and from other Canadian initiatives [15, 16] that continuing to implement, adjust, and refine a collaborative care model will positively contribute to achievement of the AHS Professional Practice Vision: *Caring, competent, committed healthcare professionals collaborating to create quality outcomes and positive patient/family experiences.* The organization is currently implementing a provincial program on over 160 units that produces tools and processes for patient-and family-centred collaborative care. The program consists of more than 20 elements, but has initially concentrated on the six elements that were implemented in the pilot unit. This study has been essential in refining the design and implementation of the first six core elements.

### Limitations

The sample size for the staff surveys was smaller than anticipated and results are subject to Type 1 error. That said, the survey results were largely echoed by the interview results, suggesting there was a positive impact on staff. This study served as an initial test of the staff survey; further validation work needs to be done in future evaluations. The same can be said of the patient surveys; a larger sample size would lend more credibility to the findings. Patient survey scores also tend to be very high in general and this ceiling effect may have reduced our ability to find meaningful change.

We also cannot generalize our findings beyond general medical units. The original intention was to include both medical and surgical units in two separate hospitals to determine whether the new processes and staffing could work in either setting, but due to various delays in implementation, only one medical unit received the full model.

## Conclusion

Overall, we found that the new model had substantially positive impacts on providers and health human resources outcomes. Although there were only a few positive effects on patient care, the fact that there were no detrimental outcomes suggests that collaborative practice models such as this one could improve AHS’s ability to deliver sustainable, high-quality, patient- and family-centred care without compromising care quality.

## Abbreviations

AHS, Alberta health services; HCA, health care aide; LPN, licensed practical nurse; NOD, name occupation duty; RN, registered nurse

## References

[1] Suter E, Deutschlander S, Mickelson G, Nurani Z, Lait J, Harrison L (2012). Can interprofessional collaboration provide health human resources solutions? A knowledge synthesis. J Interprof Care.

[2] Zwarenstein M, Goldman J, Reeves S (2009). Interprofessional collaboration: Effects of practice-based interventions on professional practice and healthcare outcomes (Review). The Cochrane Library.

[3] Berridge EJ, Mackintosh N, Freeth D (2010). Supporting patient safety: examining communication within delivery suite teams through contrasting approaches to research observations. Midwifery.

[4] Levinson W, Huynh T (2014). Engaging physicians and patients in conversations about unnecessary tests and procedures: Choosing Wisely Canada. CMAJ.

[5] Rosella LC, Fitzpatrick T, Wodchis WP, Calzavara A, Manson H, Goel V (2014). High-cost health care users in Ontario, Canada: demographic, socio-economic, and health status characteristics. BMC Health Serv Res.

[6] Alberta Health Services (2015). Alberta Health Services Health and Business Plan 2015-2018.

[7] Alberta Health Services (2013). Workforce Model Transformation Summary of Methodology and Leading Practices.

[8] Arriola KJ, Chen A, Coryell B, Shayne P, Woods A (2010). Patient and family-centred care at Emory, who cares? Current perceptions and beliefs.

[9] Braun V, Clarke V (2006). Using thematic analysis in psychology. Qual. Res. Psychol..

[10] Canadian Interprofessional Healthcare Collaborative (2010). A National Interprofessional Competency Framework.

[11] Canadian Institutes for Health Information (2014). Canadian Patient Experiences Survey – Inpatient Care.

[12] Canadian Institutes for Health Information (2014). Canadian Patient Experiences Survey – Inpatient Care: Frequently Asked Questions.

[13] Saks AM (2006). Antecedents and consequences of employee engagement. J. Manag. Psychol..

[14] Lowe G (2012). How employee engagement matters for hospital performance. Healthc Q.

[15] Fryers M, Young L, Rowland P (2012). Creating and sustaining a collaborative model of care.

[16] Tomblin Murphy G, Alder R, MacKenzie A, Rigby J (2012). Model of Care Initiative in Nova Scotia (MOCINS): Final Evaluation Report. Dalhousie University/World Health Organization Collaborating Centre on Health Workforce Planning & Research.

